# RNA Interference inhibits Hepatitis B Virus of different genotypes in Vitro and in Vivo

**DOI:** 10.1186/1471-2180-10-214

**Published:** 2010-08-10

**Authors:** Ya-Li Zhang, Tong Cheng, Yi-Jun Cai, Quan Yuan, Che Liu, Tao Zhang, De-Zhen Xia, Rui-Yin Li, Lian-Wei Yang, Ying-Bin Wang, Anthony ET Yeo, James Wai-Kuo Shih, Jun Zhang, Ning-shao Xia

**Affiliations:** 1 National Institute of Diagnostics and Vaccine Development in Infectious Diseases, School of Life Science, Xiamen University, Xiamen, Fujian Province, China

## Abstract

**Background:**

Hepatitis B virus (HBV) infection increases the risk of liver disease and hepatocellular carcinoma. Small interfering RNA (siRNA) can be a potential new tool for HBV therapy. Given the high heterogeneity of HBV strains and the sensitivity towards sequences changes of siRNA, finding a potent siRNA inhibitor against the conservative site on the HBV genome is essential to ensure a therapeutic application.

**Results:**

Forty short hairpin RNA (shRNA) expression plasmids were constructed to target conserved regions among nine HBV genotypes. HBV 1.3-fold genome plasmids carrying various genotypes were co-transfected with shRNA plasmids into either Huh7 cells or mice. The levels of various viral markers were examined to assess the anti-HBV efficacy of siRNA. Four (B245, B376, B1581 and B1789) were found with the ability to potently inhibit HBV RNA, DNA, surface antigen (HBsAg), e antigen (HBeAg) and core antigen (HBcAg) expression in HBV genotypes A, B, C, D and I (a newly identified genotype) in Huh7 cells and in mice. No unusual cytotoxicity or off-target effects were noted.

**Conclusions:**

Such siRNA suggests an alternate way of inhibiting various HBV genotypes in vitro and in vivo, promising advances in the treatment of HBV.

## Background

Worldwide, there are over 350 million people persistently infected with hepatitis B virus (HBV) [1]. Chronic HBV infections may have serious consequences, including acute hepatitis, as well as chronic hepatitis, cirrhosis, and hepatocellular carcinoma (HCC) [2]. Together, these are responsible for over 1 million deaths worldwide each year [3]. Current treatments for HBV infections are not only expensive and have significant side effects, but also only induce a partial response [4-6].

In eukaryotic cells, RNA interference (RNAi), a type of double-stranded (ds) RNA, initiates and directs sequence-specific, post-transcriptional silencing of homologous genes [7,8]. It has been demonstrated in previous studies that expression and replication of HBV can be suppressed by siRNA or shRNA with clinical implications [9-11]. However, the wide heterogeneity of HBV sequences may render RNAi inhibitors ineffective. To explore this further, 40 shRNA expression plasmids were constructed to target the sites that were conserved among HBV genotypes A through I. Their anti-HBV efficacy was then evaluated in vitro and in vivo.

## Results

### Screening for effective and broad anti-HBV shRNA

**Figure 1:**
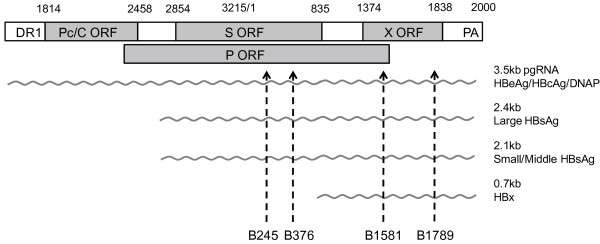
**A schematic diagram depicting the locations of siRNA targets in association with viral open reading frames and viral mRNAs within the HBV genome**. The circular HBV genome is presented in a linear form. The coding regions for e/core, surface, polymerase, and X proteins are displayed and designated as Pc/C, S, P, and X, respectively. The relative locations of the target sites of B245, B376, B1581 and B1789 are also indicated by arrowheads.

The shRNA plasmids co-transfected with two HBV 1.35 plasmids (N10 and Y1021) exhibited varying levels of extracellular HBsAg expression (Table 1). Of the forty shRNA plasmids, four plasmids (B245, B376, B1581 and B1789, Figure 1) were selected as candidates for further research based on their remarkable inhibitory ability and also relatively lower off-target probability (off-target score of above 30). The sequence conservation among the A to I genotypes for B245, B376, B1581 and B1789 were 95.1% (95%CI: 92.2~97.2), 88.7% (95%CI: 84.7~91.9), 97.3% (95%CI: 94.8~98.7), and 97.6% (95%CI: 95.2~98.9), respectively (Table 2). The data also shows that the target sequences of B245, B1581 and B1789 were more conserved than the target sequence of B376 (p < 0.05) in genotype B and C (Table 2).

**Table 1:** The characterization and screening for multiplex anti-HBV siRNA

ID	Sequence	Start Position	**Off-target number**^**a**^	**off-target score**^**a**^	Genome localization	Anti- Y1021	**Anti- N10**^**b**^
B182	GGACCCCTGCTCGTGTTACAG	182	8	30	S, P	++	+
B183	GACCCCTGCTCGTGTTACAGG	183	3	30	S, P	-	-
B184	ACCCCTGCTCGTGTTACAGGC	184	3	30	S, P	-	-
B243	AGAGTCTAGACTCGTGGTGGA	243	3	30	S, P	+	+
B244	GAGTCTAGACTCGTGGTGGAC	244	9	30	S, P	+++	+++
B245	AGTCTAGACTCGTGGTGGACT	245	4	30	S, P	+++	+++
B246	GTCTAGACTCGTGGTGGACTT	246	4	30	S, P	-	-
B250	AGACTCGTGGTGGACTTCTCT	250	10	35	S, P	-	+
B251	GACTCGTGGTGGACTTCTCTC	251	7	35	S, P	+	++
B252	ACTCGTGGTGGACTTCTCTCA	252	2	30	S, P	++	++
B375	GGATGTGTCTGCGGCGTTTTA	375	1	25	S, P	++	++
B376	GATGTGTCTGCGGCGTTTTAT	376	7	30	S, P	+++	+++
B377	ATGTGTCTGCGGCGTTTTATC	377	5	35	S, P	+	++
B379	GTGTCTGCGGCGTTTTATCAT	379	4	35	S, P	+	+
B410	ATCCTGCTGCTATGCCTCATC	410	76	25	S, P	-	-
B415	GCTGCTATGCCTCATCTTCTT	415	54	25	S, P	+	++
B456	AAGGTATGTTGCCCGTTTGTC	456	2	30	S, P	++	++
B457	AGGTATGTTGCCCGTTTGTCC	457	1	40	S, P	-	+
B458	GGTATGTTGCCCGTTTGTCCT	458	7	35	S, P	++	++
B459	GTATGTTGCCCGTTTGTCCTC	459	15	25	S, P	++	++
B461	ATGTTGCCCGTTTGTCCTCTA	461	11	30	S, P	+	+
B1260	GCCGATCCATACTGCGGAACT	1260	2	25	EnhI, P	+	++
B1577	GTGTGCACTTCGCTTCACCTC	1577	13	30	X, P, DR1	+++	++
B1579	GTGCACTTCGCTTCACCTCTG	1579	5	25	X, P, DR1	++	++
B1581	GCACTTCGCTTCACCTCTGCA	1581	15	30	X, P, DR1	+++	+++
B1583	ACTTCGCTTCACCTCTGCACG	1583	21	30	X, P, DR1	++	++
B1787	GGAGGCTGTAGGCATAAATTG	1787	4	30	Pc, EnhII	++	++
B1788	GAGGCTGTAGGCATAAATTGG	1788	9	25	Pc, EnhII	++	+
B1789	AGGCTGTAGGCATAAATTGGT	1789	5	30	Pc, EnhII	+++	+++
B1880	AAGCCTCCAAGCTGTGCCTTG	1880	3	30	Pc	+	-
B1881	AGCCTCCAAGCTGTGCCTTGG	1881	23	25	Pc	-	-
B2389	AGAAGAAGAACTCCCTCGCCT	2389	42	25	C, P	-	+
B2390	GAAGAAGAACTCCCTCGCCTC	2390	26	25	C, P	-	+
B2391	AAGAAGAACTCCCTCGCCTCG	2391	29	25	C, P	-	-
B2392	AGAAGAACTCCCTCGCCTCGC	2392	19	30	C, P	-	+
B2393	GAAGAAGAACTCCCTCGCCTC	2393	18	30	C, P	-	+
B2394	AAGAACTCCCTCGCCTCGCAG	2394	29	25	C, P	-	+
B2395	AGAACTCCCTCGCCTCGCAGA	2395	14	35	C, P	+	+
B2396	GAACTCCCTCGCCTCGCAGAC	2396	18	35	C, P	-	+
B2397	GATCCATACTGCGGAACTCCT	2397	11	35	C, P	-	-
L1254	TGGCTACATTCTGGAGACATA	NA	NA	NA	luciferase	-	-

NA, no application.

"+" indicates weak inhibition (below 50%),

"++" indicates medium inhibition (above 50%, but below 90%),

"+++" indicates strong inhibition (above 90%),

"-" indicates no significant inhibition,

An underline represents the four candidates that were worthy for further research.

^a^: off-target effects were evaluated by the online SOS program http://rnai.cs.unm.edu/offTarget.

^b^: anti-HBV effects were evaluated by decreases in extracellular HBsAg level.

**Table 2:** Sequence conservation of four selected siRNA targets in 327 HBV strains

Genotype (No. of HBV strains)	Number of strains with identical sequence with siRNA(%)				Subtype **(No. of HBV strains)**
		
	B245	B376	B1581	B2379	
Genotype A (63)	61(96.8)	62(98.4)	62(98.4)	63(100)	Aa(45), Ac(9), Ae(9)
Genotype B(72)	69(95.8)	49(68.1)*	71(98.6)	70(97.2)	Bj(9), Ba(38), B3(7), B4(8), B5(4), B6(6)
Genotype C(58)	53(91.4)	46(79.3)*	57(98.3)	56(96.6)	C1(38), C2(13), C3(2), C4(2), C5(3)
Genotype D(30)	29(96.7)	29(96.7)	28(93.3)	28(93.3)	D1(11), D2(6), D3(8), D4(5)
Genotype E(34)	33(97.1)	34(100)	33(97.1)	33(97.1)	F1(4), F2(14)
Genotype F(18)	15(83.3)	18(100)	18(100)	18(100)	
Genotype G(17)	17(100)	17(100)	15(88.2)	16(94.1)	
Genotype H(13)	13(100)	13(100)	12(92.3)	13(100)	
Genotype I(22)	21(95.5)	22(100)	22(100)	22(100)	I1(10), I2(12)
Total (327)	311(95.1)	290(88.7)*	318(97.3)	319(97.6)	

^a^: An asterisk represents a statistical difference of P < 0.05 in comparison with B376 and others.

### Adverse side-effects evaluation for selected shRNA plasmids

**Figure 2:**
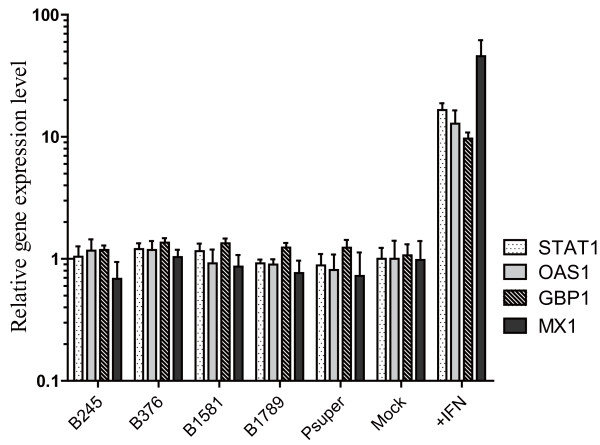
**The expression profile of four major interferon stimulated genes (ISGs) in shRNA plasmids transfected cells**. Cytoplasmic RNAs, from Huh7 cells treated with or without IFNα-2a or transfected with either pSUPER vector or shRNA plasmids, were analysed by realtime RT-PCR for IFN stimulated genes STAT1, OAS1, GBP1 and MX1. The values on the figure, plotted as "Relative gene expression level" on the y-axis, were calculated as the mRNA levels of ISGs divided by the GAPDH (control) mRNA level. Student t test was used to assess the difference between shRNA plasmids (including empty pSUPER vector) of transfected cells and non-transfected cells (mock). No significant difference was observed.

The B245, B376, B1581, and B1789 plasmids were transfected into Huh7 cells to determine cytotoxicity by the WST-8 assay. No significant siRNA-induced cytotoxicity was observed for these siRNA when compared to an empty pSUPER vector (p = 0.66, data not shown). The mRNA levels of four major interferon stimulated genes (STAT1, OAS1, GBP1 and MX1) in transfected cells were measured by quantitative realtime PCR with GAPDH mRNA acting as a control. As shown in Figure 2, between values 1 and 2, logarithmic increases for the IFN-stimulatable mRNAs were only observed in the IFN-treated cells, but not observed in any of the shRNA treated cells vs. untreated cells. From this, it can be concluded that an IFN response is not activated by these anti-HBV siRNAs.

### ShRNA inhibit gene expression of HBV strains with different genotypes in vitro

**Figure 3:**
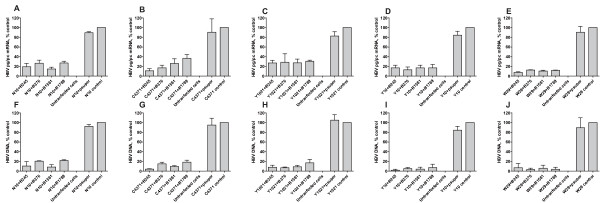
**SiRNAs inhibit RNA and DNA expression of HBV strains with different genotypes in Huh7 cells**. The histogram show the cytoplasmic HBV pg/pc RNA levels **(A, B, C, D, E) **and extracellular HBV DNA **(F, G, H, I, J) **of five HBV strains with genotypes Ae(N10), Ba(C4371), C1(Y1021), D1(Y10) and I1(W29) in treated shRNA plasmids, treated pSUPER vector, and non-treated Huh7 cells.

The levels of cytoplasmic HBV pg/pc RNA (3.5 kb) and HBV DNA in cultured supernatants were determined by realtime RT-PCR/PCR and presented in Figure 3. The pg/pc RNA level of five HBV strains with different genotypes were reduced by 58%~93% in B245(69%~93%), B376(59%~91%), B1581(67%~90%) and B1789(58%~88%) treatments, while the HBV DNA level observed in supernatants was decreased by 77%~99% in these shRNA plasmid treatments (B245: 83%~99%, B376: 79%~99%, B1581:88%~98%, B1789: 77%~99%).

**Figure 4:**
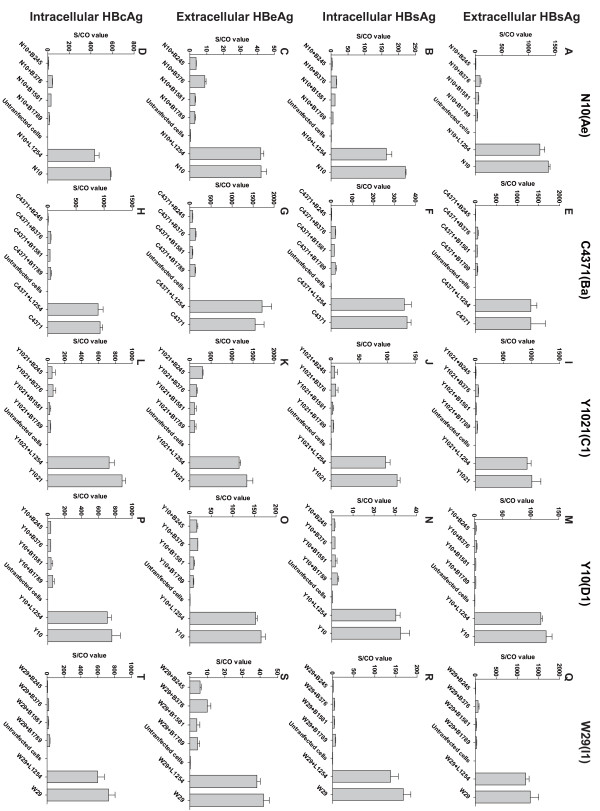
**SiRNAs inhibit viral antigens expression of HBV strains with different genotypes in Huh7 cells**. **(A, B, C, D) **Extracellular HBsAg, intracellular HBsAg, extracellular HBeAg, and intracellular HBcAg expression levels of HBV N10(Ae), respectively. **(E, F, G, H) **Extracellular HBsAg, intracellular HBsAg, extracellular HBeAg, and intracellular HBcAg expression levels of HBV C4371(Ba), respectively. **(I, J, K, L) **Extracellular HBsAg, intracellular HBsAg, extracellular HBeAg, and intracellular HBcAg expression levels of HBV Y1021(C1), respectively. **(M, N, O, P) **Extracellular HBsAg, intracellular HBsAg, extracellular HBeAg, and intracellular HBcAg expression levels of HBV Y10(D1), respectively. **(Q, R, S, T) **Extracellular HBsAg, intracellular HBsAg, extracellular HBeAg and intracellular HBcAg expression levels of HBV W29(I1), respectively.

**Figure 5:**
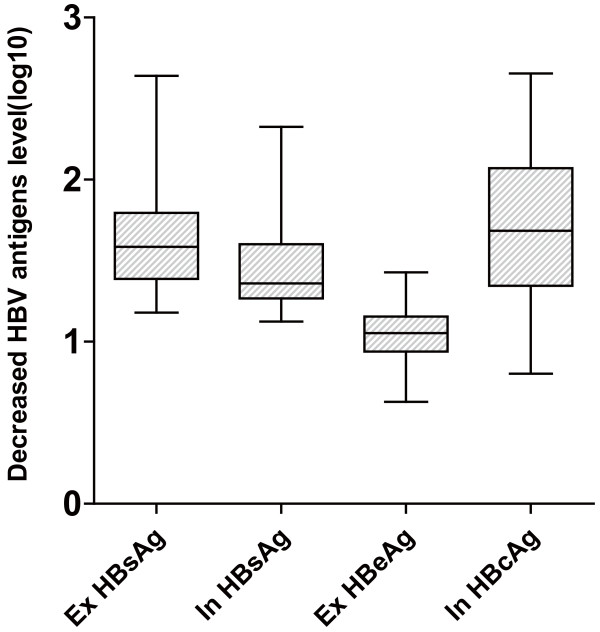
**Comparing the RNAi-induced silencing effect on different viral markers**. Data were displayed the average antigen level of the 4 siRNAs reduced for five HBV strains. "Ex" = Extracellular and "In" = Intracellular. The Mann-Whitney test was used to assess the difference. An asterisk represents a statistical difference of P < 0.01 in comparison with the other markers (Ex HBeAg vs. Others P < 0.001, Ex HBsAg vs. In HBsAg P = 0.05, Ex HBsAg vs. In HBcAg P = 0.82, In HBsAg vs. In HBcAg P = 0.10.)

In addition, the extracellular and intracellular antigen levels in Huh7 cells that were co-transfected with HBV and shRNA plasmids were also determined (Figure 4). In the shRNA-treated Huh7 cells, the average extracellular HBsAg expression level of all five HBV strains decreased by 1.66 ± 0.36 logs. The average intracellular HBsAg expression level decreased by 1.47 ± 0.33 logs, while the extracellular HBeAg levels decreased by 1.04 ± 0.23 logs, and the intracellular HBcAg levels by 1.71 ± 0.49 logs. The effect of the siRNA treatment on HBeAg levels was weaker than that on the HBsAg or HBcAg levels (P < 0.001, Figure 5).

### Inhibition of gene expression of HBV strains with different genotypes in vivo

**Figure 6:**
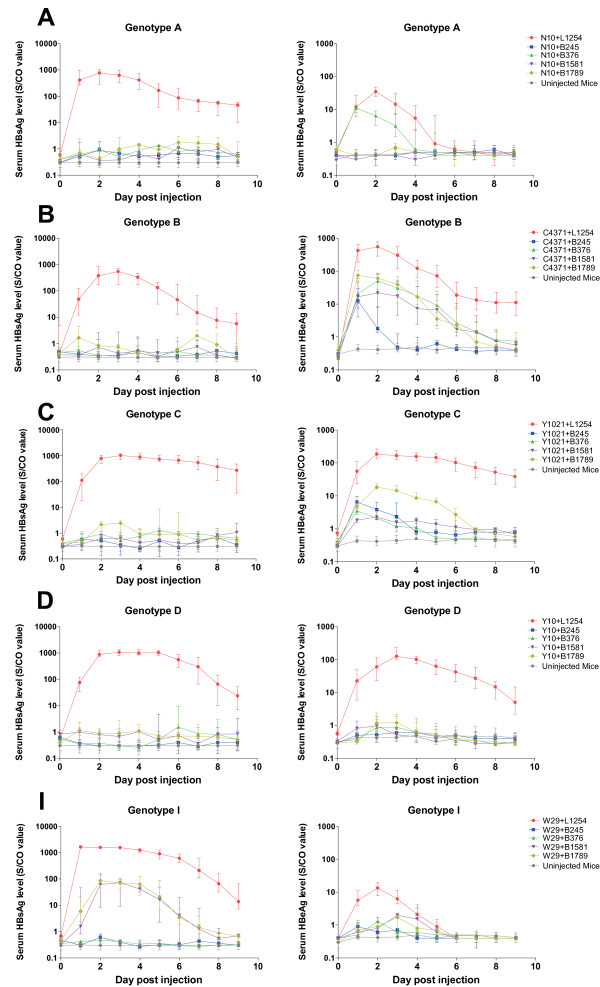
**Kinetics of serum HBV antigen (HBsAg and HBeAg) of various HBV genotypes in RNAi-treated mice**. For each group (each line in the figure), the experiment was repeated using two different groups of five mice. Due to limited serum resources, each sample was diluted 10-fold. **(A) **Genotype Ae (N10 group), **(B) **Genotype Ba (C4371 group), **(C) **Genotype C1 (Y1021 group), **(D) **Genotype D1 (Y10 group), **(E) **Genotype I1 (W29 group).

Using the mouse model of acute hepatitis B virus infection [12], the profiles of serum HBsAg and HBeAg were used to evaluate the effect of shRNA over nine days (Figure 6). All HBV plasmids expressed detectable HBsAg and HBeAg in mice sera (Figure 6). As compared to the control mice (HBV+L1254), B245 and B376 treatments reduced HBsAg expression by over 99% in all five HBV genotypes. Furthermore, B1581 and B1789 treatments suppressed HBsAg by over 99% in mice infected with HBV genotypes A, B, C and D. In a novel W29 strain representing genotype I however, B1581 and B1789 treatments only reduced HBsAg expression by about 90%. With regards to serum HBeAg for genotypes A, B, C, D and I, B245, B376, B1581 and B1789 treatments suppressed HBeAg by 96%~99%, 79%~99%, 94%~99%, and 89%~99%, respectively. The overview of the results shows that B245 is the most potent agent.

## Discussion

Activated RNAi pathway can silence HBV replication and expression [13,14]. However, in most previous studies, the activity of RNAi against HBV is often evaluated with only one HBV strain [15-18]. Nine HBV genotypes (including a newly identified genotype "I"), designated as the letters A through I, have been recognized with an accompanying sequence divergence of >8% over the entire genome [19-21]. The influence of genotypes on HBV replication efficacy and antigen expression level had been proved to be various and that may further associate with clinical outcomes and antiviral treatments responses [22]. Hence, RNAi designed for one genotype may not necessarily be effective against another genotype. Given the high heterogeneity of HBV strains and the sensitivity of siRNA to the sequence changes, designing siRNA targets against the conservative site on HBV genome is essential to ensure activity across all genotypes [23].

In shRNA expression systems, two different promoters are predominantly used: U6 and H1, both driven by human polymerase III (poly III). Compared to Pol II promoters, Pol III promoters generally possess a greater capacity to synthesize RNA transcripts of a higher yield and rarely induce interferon responses [17,24]. However, a previous study noted that U6 Pol III-expressed shRNAs may cause serious toxicity in vivo by saturating the endogenous miR pathway [25]. In this report, we constructed 40 shRNA plasmids (Table 1) with various targets, using a human H1 Pol III promoter. The target sequences of the final four selected shRNA plasmids demonstrated high sequence conservation among A to I genotypes and significant inhibition activity, in both Huh7 cells and mice, against the expressions of HBV RNA, DNA and antigens in genotypes A, B, C, D and I. The inhibitory efficacy of these shRNAs (B245, B376, B1581 and B1789) however, varies significantly against the various genotypes for different viral markers in different models (Figure 3, 4, 5 and 6). Such differences in efficiency may be due to differences in the mRNA's secondary structure or the target site accessibility [26]. B245 was the most effective of the four candidates.

It should be noted that both the cell-transfection model and hydrodynamic injection model more closely resemble an acute model of a HBV infection. This is a potential limitation in this study, as most individuals who need anti-HBV therapy are chronically infected. Compared to the HBV transgenic mouse models and stably transfected cell lines, the former are more flexible and convenient in evaluating the efficacy of shRNAs as a way to inhibit various HBV strains. Nevertheless, the effective shRNA candidates should be studied further in different models.

Because HBV contains overlapping open reading frames (ORFs) and all four HBV transcripts overlap in their 3' terminals, a single siRNA targeting multiple areas could be designed to maximize inhibitory potency [23]. The siRNAs targeting C ORF, such as B2389~B2397, presented in Table 1, show activity only against the 3.5 kb pregenomic RNA, but are unlikely to show any activity against the other three transcripts (Figure 1). Meanwhile, all four siRNAs demonstrated more silencing activity with regards to HBsAg expression than HBeAg expression for various genotypes in the cell cultures and mice. The targets on both however were the same in the HBV transcripts for the two proteins (Figure 4 and Figure 5), which was also observed in a previous study [23]. HBcAg, a viral capsid correlated with viral replication [27,28], was silenced as effectively as HBsAg, but HBeAg was not (Figure 4).

The registered agents currently available for the treatment of HBV infections, such as interferon and nucleoside analogues, can dramatically decrease HBV DNA levels and induce particular HBeAg loss, but will rarely cause HBsAg loss in chronic hepatitis B patients [29-32]. RNA interference, on the other hand, can theoretically be directed to cleave any target RNA, providing a novel methodology for anti-HBV therapy [33]. In the present study, and supported by other studies [13,34,35], using RNAi as an inhibitor for HBV effectively reduces viral antigen levels, including HBsAg. It can be speculated that RNAi-treatments may offer complementary effects for current anti-HBV therapy. However, the final application of RNAi-based anti-HBV drugs depends on the development of effective and safe RNAi delivery systems.

## Conclusions

In summary, four candidate shRNA plasmids significantly inhibited HBV genotypes A, B, C, D and I in vitro and in vivo. A potential avenue of investigation would be a combination strategy of various siRNA in a single transcript to improve efficacy and also prevent or at least delay the rise of viral escape mutants.

## Methods

### HBV Plasmids

Five HBV 1.35-fold genome plasmids - N10 (genotype Ae, AY707087), C4371 (genotype Ba, GU357842), Y1021 (genotype C1, GU357845), Y10 (genotype D1, GU357846) and W29 (genotype I1, GU357844) were used for transfection and hydrodynamic injection. The constructions and molecular and phenotypic characteristics are described in our previous report [36].

### Bioinformatics Analysis

To define the conservative sites on HBV genomes amongst the various genotypes, all available complete genome sequences of HBV, as of April 2009, were downloaded from GenBank. Multiple alignment was done with ClustalX2 under default settings (Gap Opening:10, Gap Extension: 0.2, Delay Divergent Sequences(%): 30, DNA Transition Weight: 0.5, Use Negative Matrix: Off). The most representative and informative sequence in terms of phylogeny were collected as a dataset and the most similar sequences were removed using all pairwise distance scan. A total of 327 HBV genomes including A-I genotypes and nearly all reported subtypes were remained in the final dataset. The genotypes and subtypes of six HBV genomes isolated in the study were submitted to phylogenetic analysis using MEGA 4.0 software (data not shown). Forty sites with conservative sequences were selected and the shRNA plasmids were constructed (Table 1). The designed siRNA were evaluated for potential off-target effects by the online SOS program http://rnai.cs.unm.edu/offTarget. The sequences and positions of the forty designed shRNA targets are shown in Table 1.

### ShRNA Plasmids

ShRNA plasmids were cloned downstream of the human H1 promoter in the vector pSUPER [37]. The target sites for siRNA were chosen based on conservative sites among the major HBV genotypes and subtypes. An shRNA plasmid targeting the firefly luciferase gene was used as a control (L1254: TGG CTA CAT TCT GGA GAC ATA).

### Cell Culture and In Vitro Transfection

The plasmids used for *in vitro *transfection were purified with PlasmidSelect Xtra Starter Kit (GE Health, Sweden) and the concentrations were determined by the UV-spectrophotometric method. To determine the ability of siRNA to inhibit HBV gene expression in cell cultures, Huh7 cells were co-transfected with 4 μg of HBV plasmids, 1 μg of shRNA plasmids and 0.4 μg of a pcDNA3.1-SEAP plasmid using Lipofectamine 2000 (Invitrogen, Shanghai, China) following the manufacturer's instructions. They were then harvested four days later. The pcDNA3.1-SEAP plasmid is a reporter plasmid expressing secreted alkaline phosphatase and used for transfection efficiency standardization by estimating SEAP enzymatic activity (Pierce; Kunming, China) in the culture supernatant.

### Evaluation for Potential Adverse Effects of siRNA

Possible adverse effects of shRNA on cells were evaluated using morphology criterion, growth rate assessment, and by noting the cytotoxicity profile of transfected cells. Cytotoxicity was determined through a WST-8 assay (Cell Counting Kit-8, Beyotime, Shanghai, China) [38,39]. The number of viable cells was then determined by absorbance measured at 450 nm on an automated plate reader. The potential off-target effects of siRNA were evaluated by monitoring the IFN response. Huh7 cells were transfected with 1 μg of shRNA plasmids. Non-transfected cells treated or untreated with 500 IU of IFNα-2a (Anfulong, Huadali Company, China) for 24 h served as a positive control [40]. Expression profile of four major interferon-stimulated (STAT1, OAS1, GBP1 and MX1) were analyzed by a quantitative RT-realtime PCR using the previously reported primers while the GAPDH level served as a control[41].

### Mice Experiments

To evaluate the anti-viral effects of siRNA in vivo, an HBV hydrodynamic injection was conducted in BALB/c mice. Briefly, 50 μg of purified HBV plasmid and 10 μg shRNA plasmids were diluted to 2 mL with physiological saline and then injected into the tail vein within 5-10 s. Mice sera were assayed every day for HBsAg and HBeAg from Day 0 to Day 9. For each group, five mice aging from 4-6 weeks were used [42]. All animals received humane care and the study protocol complied with the institution's ethics guidelines.

### Measurement of HBV RNA and DNA

For detection of the cytoplasmic HBV RNA, total RNA was extracted from cells using Tripure Isolation Reagent (Roche Applied Science, Switzerland) according to the manufacturer's instructions. Potential residual DNA contamination of RNA preparations were excluded by DNase I digestion. Ten nanograms of RNA were analysed by AccessQuick realtime RT-PCR System (Promega, USA) on a CFX96 instrument (Bio-Rad, USA). The HBV pg/pc (pregenomic/preCore) RNA level was detected by primers PGP (-CACCTCTGCCTAATCATC, nt1826-nt1843) and BC1 (GGAAAGAAGTCAGAAGGCAA, nt1974-nt1955) [43] using probe CP2 (HEX-ATGTTCATGTCCTACTGTTCAAGCC-BHQ2). The transcript copy number was normalized to those of GAPDH.

For the HBV DNA assay, 100 μL of supernatant was pre-heated at 50°C for 20 minutes and then treated with 1 U DNase I for 2 hours to eliminate residual plasmids. The reaction was terminated by EDTA at a final concentration of 10 mM. The mixture was then incubated at 70°C for 10 min and the HBV DNA was extracted using QIAamp DNA blood kits (QIAGEN, Hilden, Germany). HBV DNA quantification assays were performed using a commercial real-time PCR kit (Kehua, Shanghai, China).

### Determination of HBV Antigens

HBsAg, HBeAg and HBcAg levels were determined by chemiluminescence using commercial assay kits (Wantai, Beijing, China). The relative level of each antigen was expressed as an S/CO (signal/cutoff) value, on a linear range from 1 to 1000 for all three assays. The lower detection limit was 10 pg/mL for the HBsAg and HBeAg assays, and 50 pg/ml for the HBcAg assay. In regards to the intracelluar HBV antigen assay, the transfected cells were treated with a suitable lysis buffer (20 mM HEPES, 1 mM EGTA, 100 mM NaCl, 5 mM Mg_2_Cl, 0.4% n-Dodecyl β-D- maltoside, n-Dodecyl β-D-maltoside, and 10% Glycerol) at room temperature for 30 minutes and the supernatants were separated through centrifugation and used for immunoassay.

### Statistical Evaluation

Statistical analyses were performed using independent Student t test or Mann-Whitney U test (GraphPad Software, San Diego California USA,). Differences were considered to be statistically significant for p values ≤ 0.05.

## Abbreviations

**SIRNA**: small interfering RNA; **SHRNA**: short hairpin RNA; **OFF-TARGET EFFECT**: non-specific effects resulting from the introduction of siRNA; **STAT1**: signal transducers and activators of transcription1; **OAS1**: 2'-5'-oligoadenylate synthetase 1; **MX1**: interferon-induced GTP-binding protein; **GBP1**: guanylate binding protein 1; **HBV**: hepatitis B virus; **HBSAG**: hepatitis B surface antigen; **HBEAG**: hepatitis B e antigen; **HBCAG**: hepatitis B core antigen.

## Authors' contributions

YLZ, TC, JZ and NSX conceived the study, participated in its design and coordination and drafted the manuscript. YLZ and QY carried out the molecular genetic studies, analyzed the aligned sequences, found conserved targets, participated in the study design and were involved in the shRNA design. YZL and YJC constructed all shRNA plasmids. YZL, YJC, CL, TZ, DZX, RYL, LWY and YBW performed all cell and mice experiments (including all transfections, hydrodynamic injections, WST-8 assays, RT-PCR and chemiluminescence immunoassays). YLZ, YJC, TC and QY conducted the data analysis and interpretation. AEY, JWS, QY, JZ and NSX helped to draft the manuscript and critically revised its final version. TC, JZ and NSX obtained funding. All authors read and approved the final manuscript.
